# Does Elite Sport Degrade Sleep Quality? A Systematic Review

**DOI:** 10.1007/s40279-016-0650-6

**Published:** 2016-11-29

**Authors:** Luke Gupta, Kevin Morgan, Sarah Gilchrist

**Affiliations:** 1 Physiology Department, English Institute of Sport, Bisham, Nr. Marlow, SL7 1RR UK; 2 Clinical Sleep Research Unit, School of Sport, Exercise and Health Sciences, Loughborough University, Loughborough, LE11 3TU UK

## Abstract

**Background:**

Information on sleep quality and insomnia symptomatology among elite athletes remains poorly systematised in the sports science and medicine literature. The extent to which performance in elite sport represents a risk for chronic insomnia is unknown.

**Objectives:**

The purpose of this systematic review was to profile the objective and experienced characteristics of sleep among elite athletes, and to consider relationships between elite sport and insomnia symptomatology.

**Methods:**

Studies relating to sleep involving participants described on a pre-defined continuum of ‘eliteness’ were located through a systematic search of four research databases: SPORTDiscus, PubMed, Science Direct and Google Scholar, up to April 2016. Once extracted, studies were categorised as (1) those mainly describing sleep structure/patterns, (2) those mainly describing sleep quality and insomnia symptomatology and (3) those exploring associations between aspects of elite sport and sleep outcomes.

**Results:**

The search returned 1676 records. Following screening against set criteria, a total of 37 studies were identified. The quality of evidence reviewed was generally low. Pooled sleep quality data revealed high levels of sleep complaints in elite athletes. Three risk factors for sleep disturbance were broadly identified: (1) training, (2) travel and (3) competition.

**Conclusion:**

While acknowledging the limited number of high-quality evidence reviewed, athletes show a high overall prevalence of insomnia symptoms characterised by longer sleep latencies, greater sleep fragmentation, non-restorative sleep, and excessive daytime fatigue. These symptoms show marked inter-sport differences. Two underlying mechanisms are implicated in the mediation of sport-related insomnia symptoms: pre-sleep cognitive arousal and sleep restriction.

**Electronic supplementary material:**

The online version of this article (doi:10.1007/s40279-016-0650-6) contains supplementary material, which is available to authorized users.

## Key Points

**Table:** 

Insomnia symptomatology is high among elite athletes, with sleep quality appearing most vulnerable prior to major competitive events, during periods of high-intensity training and following long-haul travel to competitions.
Athlete sleep disturbances can affect training and competition directly, through fatigue, or indirectly, through sleep-related performance anxieties.
In general, the quality of the evidence base addressing sleep quality among elite athletes is low, with poor operationalisation of sleep quality constructs and few controlled comparisons of athlete and non-athlete sleep.

## Introduction

While reviews of the sport [1–9] and exercise [9–13] literature suggest a reciprocal relationship between sleep and athletic performance, this evidence provides an incomplete indication of relevant sleep–sport interactions. To date, most attention has focussed on relationships between athletic performance and either the electrophysiological composition and duration of sleep or, more recently, actigraphic measurements of sleep–wake patterns [14]. Much less attention, however, has been paid to interactive associations between sleep *quality* (i.e. the subjective experience and perceived adequacy of sleep), and the demands of elite sport participation. As a result, information on insomnia symptomatology and its implications for sports performance among elite athletes remains poorly explored, and poorly systematised.

Insomnia is defined by the cardinal symptoms of difficulty initiating or maintaining sleep (despite adequate opportunity *to* sleep), and/or non-restorative (unrefreshing) sleep, together with impaired daytime functioning (International Classification of Sleep Disorders, ICSD [15]). Daytime impairments can range from manifest fatigue [15] and emotional dysregulation [16] to more subtle deficits in psychomotor [17] and neuropsychological [18] performance. Importantly, the symptom of excessive daytime sleepiness (EDS), as opposed to fatigue, is not a typical characteristic of insomnia; objective (e.g. multiple sleep latency tests, which directly assess daytime sleep pressure) [e.g. 19, 20], and subjective measures of daytime sleepiness [e.g. 20] poorly discriminate between those with insomnia and controls. Research evidence supports the view that insomnia is a disorder of hyperarousal, where the healthy transition from wake to sleep is substantially inhibited by two processes: (1) ‘cognitive arousal’ [21, 22], engagement in seemingly uncontrollable pre-sleep cognitive activity which ultimately triggers physiological (autonomic, cortical, metabolic) responses inconsistent with pre-sleep de-arousal; and (2) ‘attentional bias’ [22], a tendency to focus excessively on [23], or a difficulty in switching attention from [24] sleep-related problems. The research evidence also suggests that personality attributes, particularly those reflecting ‘anxious concerns’ or traits associated with perfectionism [25], and difficulties in regulating arousal, can combine in certain ‘at risk’ phenotypes, making some people inherently more predisposed to insomnia than others [26].

In the context of elite sport, these characteristics of insomnia symptomatology introduce a range of clinical, empirical and theoretical factors which justify specific attention to sleep quality (in addition to sleep structure and patterns) when reviewing sleep and sport interactions.

1. In previous reviews [9–13], the impact of exercise on sleep has most frequently been expressed in terms of post-exercise changes in sleep-stage organisation, with increases in levels of exercise leading to greater duration and intensity of stage 3 sleep [9–13]. It has long been recognised, however, that polysomnographic macrostructure (as reflected in standard sleep stages) poorly discriminates between those who report good quality sleep and those who report symptoms of insomnia (e.g. Edinger et al. [27]), with evidence suggesting that any differences may reside more in polysomnographic microstructure [18] or, where subjective estimates of sleep duration are concerned, in psychological characteristics which mediate sleep experience [28]. Previous emphasis on sleep structure and exercise, therefore, inadequately addresses issues of sleep quality.
2. Sleep and sport [1–7] reviews have typically equated exercise-training-related sleep disturbance with sleep *loss*, exploring the impact of disordered sleep on athletic performance through sleep deprivation models (e.g. see Fullagar et al. [29]). However, since insomnia *per se* is not characterised by EDS [19], such models may provide limited insights into *sleep quality*–performance relationships (a point recently emphasised in the sports literature by Dickinson and Hanrahan [30]).
3. Elite athletes are rigorously selected on the basis of not only physiological but also psychological [31] attributes. It is possible that some of the personality (and relevant genetic) characteristics which militate towards success within elite sport (i.e. perfectionism and anxious concern) also predispose individuals to insomnia (e.g. see Harvey et al. [26]).
4. For elite athletes, the multifaceted demands of elite sport, including the heightened frequency, intensity and volume (and also scheduling [32, 33]) of training sessions [34–36], pre-competition anxiety (a type of cognitive arousal) [37, 38] and the relocation required by national [39, 40] and international competitions [41, 42], can all be expected to precipitate (or perpetuate) episodes of sleep disruption. Chronic sleep disturbances [43, 44], in addition to restricted sleep times [32, 33], may contribute to the high levels of daytime fatigue typically reported by competitive athletes and thus may impair training quality and adherence. The degree of sleep disruption and daytime symptoms (i.e. manifest fatigue) will be amplified in predisposed individuals.

### Objectives

To date, no attempt has been made to systematise and critique the literature on sleep quality in relation to elite sport. Given this, the present review was designed to achieve two aims: (1) to assess the structure, patterns and quality of sleep in elite athletes; and (2) to consider specific risk factors for sleep disturbance arising from the demands of elite sport. In light of points 1–4 (above), the working hypothesis was that elements of elite sport will negatively impact on sleep quality in elite athletes.

## Methods

**Fig. 1:**
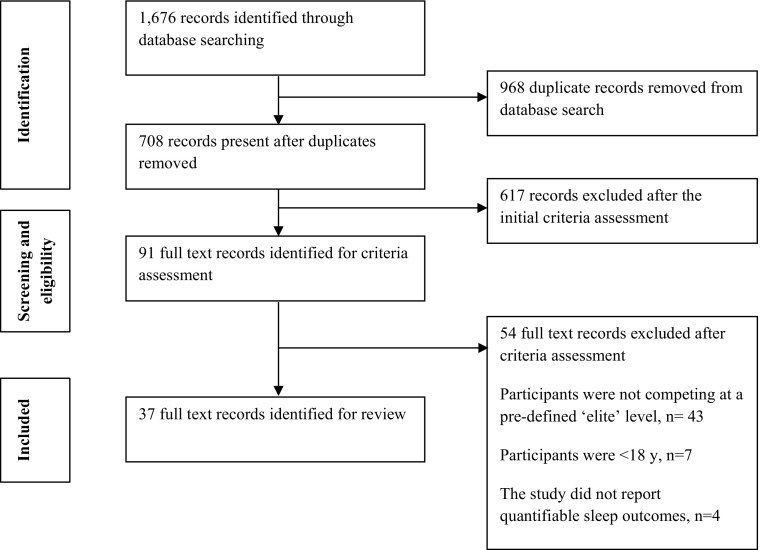
Study selection PRISMA flow diagram

The review methodology adopted the Preferred Reporting Items for Systematic Reviews and Meta-Analyses (PRISMA) guidelines. The search strategy, together with the number of hits at each stage, is shown in Fig. 1.

### Search Strategy

Four electronic databases (SPORTDiscus, PubMed, Science Direct and Google Scholar) were systematically searched up to April 2016 using combinations of the following key words with appropriate truncation and medical subject headings (MeSH): sleep, sleep quality, insomnia, elite athletes, high-performance athletes, training, travel, competition and recovery.

### Eligibility Criteria

The researchers independently assessed the eligibility of retrieved records on the basis of title and abstract. If the information was unclear, the full-text article was screened. Studies were required to meet the following inclusion criteria: (1) data were reported for participants competing at the elite level (defined as Olympic, international, professional or national); (2) data were reported for participants aged ≥18 years; (3) the study reported quantitative data on sleep outcomes; and (4) the study was published in a peer-reviewed journal as a full-text article.

### Study Selection and Data Extraction

Titles and abstracts of potentially relevant articles were screened independently by two reviewers (LG and KM). Duplicates were removed and articles which did not meet the inclusion criteria were excluded. Full-text articles were assessed for eligibility by two reviewers (LG and KM). A pre-designed data extraction form was employed to collate data from individual studies including study design, participant ‘eliteness’ (see Sects. 2.4, 3.3), *n* size, participant sex, methodology of sleep assessment, and key outcomes and findings.

### Study Quality Appraisal

Studies were critically appraised for evidence quality (principally participant selection, comparability and outcomes) using the Newcastle-Ottawa Scale (NOS) adapted for cross-sectional studies (see Electronic Supplementary Material Table S1). This scale has been employed in systematic reviews of elite athletes [45] and has established content and inter-rater reliability. Participant ‘eliteness’ for included studies was judged by applying the taxonomy of Swann et al. [46], which ranks participants on a continuum (score range 1–16), allowing categorisations from ‘semi-elite’, through ‘competitive elite’, and ‘successful elite’, to ‘world-class elite’ (see Electronic Supplementary Material Table S2).

### Definitions of Sleep Terminology

Definitions of the key sleep-related terms used in this review are shown in Table 1. In brief, ‘sleep quality’ refers to subjective sleep experience quantified using global items or formal psychometric assessments; ‘sleep pattern’, on the other hand, refers to serial instrumental measurements of 24-h sleep–wake distributions (typically using actigraphy). For polysomnographic (PSG) studies, ‘sleep structure’ refers to the organisation of sleep stages within a recorded sleep phase. It should be noted that scoring criteria for classifying sleep stages changed in 2004 when the five-fold (stage 1, stage 2, stage 3, stage 4 and REM—rapid eye movement sleep) classification of Rechtschaffen and Kales [47] was replaced by the American Academy of Sleep Medicine classification: stage N1 (stage 1); stage N2 (stage 2); stage N3 (stages 3 and 4); and stage R (REM) [48].

**Table 1:** Sleep-related terms used in this review

Term	Definition
Sleep structure	The electrophysiological composition and organisation of sleep typically described in terms of the duration of defined sleep stages, the duration of the awake stage, the aggregated time spent in all sleep stages (i.e. TST), or latencies to sleep onset (see below) and other (e.g. rapid eye movement sleep onset latency) stages. When combined with measures of TIB, electrophysiogical measures can provide a reliable indication of SE (see below). Such electrophysiological measures require polysomnography (PSG), and are rarely used for 24-h or serial measurements
Sleep patterns	As used in this review, ‘sleep patterns’ refer to sleep–wake distributions typically assessed over units of 24 h using wrist actigraphy (and often repeated for multiple days). When combined with measures of TIB, actigraphy can provide a reliable indication of SL and SE (see below)
Sleep quality	An individual’s subjective experience of sleep typically focussing on problems initiating or maintaining sleep, or early morning awakening. Assessed through single items or formal psychometric evaluations, these experiences represent cardinal symptoms of insomnia. Combined with information on symptom frequency/duration and daytime symptoms (e.g. fatigue), these experiences contribute to diagnostic judgements of insomnia *disorder* as defined in Diagnostic and Statistical Manual of Mental Disorders, fifth edition (DSM-V) [49]
Sleep profile	Broadly, SOL, TST, and SE reported
Time in bed (TIB)	The time elapsed between first getting into bed (with the intention of sleeping), to the final arising
Sleep period time (SPT)	The time elapsed between the first onset of sleep and the final awakening
Wake after sleep onset (WASO)	The amount of wakefulness accumulated between the first onset of sleep and the final awakening
Total sleep time (TST)	The total amount of time spent asleep whilst in bed (i.e. SPT-WASO)
Sleep efficiency (SE)	TST expressed as a percentage of TIB: TST/TIB × 100. Whether derived from instrumental measures or subjective estimates (of TST), SE provides a sensitive metric for estimating sleep quality. A SE below 85% is indicative of disorder
Sleep onset latency (SOL) or sleep latency (SL)	The time elapsed between getting into bed or ‘lights out’ to sleep onset
Fragmentation index	A measure of the extent to which continuous sleep is interrupted by episodes of wakefulness. Sleep fragmentation is reflected in the duration and/or frequency of episodes of WASO

## Results

### Study Selection

The search strategy returned 1676 records. Of the 91 studies retained for full-text screening, we excluded 54 which did not meet the set criteria. Thirty-seven studies were therefore eligible for review.

### Characteristics of Included Studies

The 37 studies were published between 2001 and 2016 and included Olympic/Commonwealth (*n* = 21, 57%), Paralympic (*n* = 4, 11%), and professional (*n* = 12, 32%) sports. The number of participants in the included studies ranged from 6 to 2067, with an overall age range of 18–30 years. Female athletes were under-represented generally, with only eight (21%) of the studies reporting values exclusively for women.

### ‘Eliteness’ of Athletes

The application of the full Swann et al. [46] taxonomy was limited by participant descriptions within the included studies. Only a minority of studies (*n* = 6, 16%) reported ‘athlete level of experience’, with only one study reporting ‘athletes’ level of success’. As a result, participants were categorised using a modified taxonomy within which only ‘semi-elite’ or ‘competitive elite’ categories could be judged (see Electronic Supplementary Material Table S2). Accordingly, 20 studies (54%) were judged to have recruited ‘competitive elite’ participants.

### Evidence Quality Appraisal

The evidence quality appraisal of the 37 studies can be seen in Electronic Supplementary Material Table S1. Given the diversity of study designs, reporting standards, outcome variables, and the very limited number of control participants, a meaningful calculation of risk (as odds ratios) and a subsequent meta-analysis was not possible. However, where studies reporting sleep quality outcomes used similar instruments, pooled estimates of prevalence were calculated. Relative to the objectives of this review, studies fell into three categories: (1) studies describing sleep structure and patterns; (2) studies describing sleep quality and insomnia symptomatology; and (3) studies exploring sport-related risk for sleep disturbances.

Overall, evidence quality of the selected studies was generally ‘low’ (mean NOS score = 5, standard deviation [SD] = 2), with 23 studies (62%) scoring <5 (low quality) and only two (5%) scoring >7 (good quality). Study designs employed were generally observational (*n* = 34, 92%), with only three (8%) of this sample employing control-group designs. Of the observational studies, 18 (49% of all studies) were cross-sectional and 14 (38% of all studies) were longitudinal. Very few studies (14%, *n* = 5), adequately reported participant level of performance, age, sex, sport, and level of experience. Just under half of the studies (49%, *n* = 18) provided a clear description of the protocol employed to measure sleep and used validated instruments, whilst adhering to measurement standards.

### Sleep Structure and Patterns

A total of 20 studies, published between 2001 and 2015, describing typical sleep profiles, assessed using wrist actigraphy (*n* = 11), PSG (*n* = 2), sleep diaries (*n* = 4) and questionnaires (*n* = 4), for mainly male elite athletes engaged in *normal* training are shown in Table 2. Earlier studies showed a preference for PSG, while the more recently published studies utilised wrist actigraphy and self-report inventories principally focussing on total sleep time, sleep efficiency and sleep onset latency. Only one actigraphy study compared athletes with controls. Leeder and colleagues [50] found no significant difference in total sleep time between 46 Summer Olympic athletes and 20 age-matched non-athletes, but did report a significantly lower sleep efficiency, and significantly higher time in bed, wake time after sleep onset, sleep onset latency and sleep fragmentation in athletes. Differences between sports were reported in three actigraphy studies [32, 50, 51]. In a comparison of team and individual sports [51], individual competitors showed significantly lower total sleep times and sleep efficiencies, and longer sleep latencies. On the other hand, a comparison of canoeing, diving, rowing and skating athletes reported the lowest total sleep times, the shortest sleep onset latencies, but the highest sleep efficiencies for rowers [50]. Consistent with these findings, individual sports were reported to have shorter total sleep times than team sports and napped more frequently in the day (15% of 754 days) than team sports (11% of 613 days) [51]. In the only study to compare sex [50], the time in bed of male athletes was reported to be 54 min longer than that for female athletes.

**Table 2:** Sleep characteristics of elite athletes

Study	Sport	Level of performance description	*n*	Sex	Nights recorded	Mean values (SD)			
						Age, y	TST, h	SE, %	SOL, min
Actigraphy									
Leeder et al. [50]	Multi-sports	GB squad	46	M + F	4	NR	6.9 (0.7)	81 (6)	18 (17)
Lastella et al. [51]	Multi-sports	Elite	124	M + F	12	22.2 (3.0)	6.8 (1.2)	86 (6)	19 (24)
Richmond et al. [40]	ARF	Professional	19	M	4	24.1 (3.3)	8.9 (0.1)	93 (1)	NR
Richmond et al. [39]	ARF	Professional	10	M	5	23.0 (2)	8.4 (0.3)	88 (4)	NR
Romyn et al. [52]	Netball	State level	8	F	7	19.6 (1.5)	8.2 (0.5)	85 (4)	28 (26)
Schaal et al. [53]	SS	International	10	F	7	20.4 (0.4)	7.2 (0.2)	85 (1)	17 (2)
Sargent et al. [32]	Multi-sports	Elite	70	M + F	14	20.3 (2.9)	6.5 (1.5)	86 (7)	NR
Robey et al. [54]	Football	Elite youth	12	M	12	18.5 (1.4)	7.2 (0.7)	89 (6)	21 (11)
Sargent et al. [55]	Cycling	National	16	M	8^a,b^	19.3 (1.5)	7.6 (0.6)	85 (5)	17 (14)
Shearer et al. [56]	Rugby Union	Elite	28	M	4	24.4 (2.9)	7.1 (1.0)	79 (9)	34 (40)
Lastella et al. [57]	Football	Elite	16	M	3	18.8 (0.9)	7.5 (1.3)	85 (NR)	NR
Mean					7 (4)	21.0 (2.2)	7.5 (0.7)	86 (5)	22 (7)
Sleep diary									
Fullagar et al. [58]	Football	Elite	15	M	3	25.5 (4.9)	8.5 (1.2)	92 (4)	20 (17)
Fullagar et al. [59]	Football	Elite	16	M	NR	25.9 (7.5)	8.7 (0.7)	96 (NR)	16 (7)
Kölling et al. [60]	Rowing	National	55	M + F	6	17.7 (0.6)	6.9 (0.4)	93 (4)	26 (17)
Fowler et al. [42]	Rugby league	Professional	18	M	1	24.2 (3.3)	7.9 (1.0)	NR	NR
Mean						23.3 (3.8)	8.0 (0.8)	94 (2)	21 (5)
Polysomnography									
Sargent et al. [55]	Cycling	National	16	M	8^a^	19.3 (1.5)	8.5 (0.4)	90 (5)	18 (13)
Netzer et al. [61]	Cycling	National	13	M	1	23.9 (NR)	NR	93 (3)	19 (16)
Mean						21.6 (3.3)	8.5 (NA)	92 (2)	19 (1)
Questionnaire^c^									
Tsunoda et al. [62]	WCB	Elite	14	M	NA	29.5 (5.2)	6.5 (0.9)	88 (9)	25 (22)
Swinbourne et al. [63]	Team sports	National	175	M + F	NA	21.9 (2.6)	7.9 (1.3)	NR	NR
Bleyer et al. [64]	Multi-sports	Elite	452	M + F	NA	21.2 (5.8)	7.9 (1.5)	NR	NR
Durán et al. [65]	Multi-sports (Paralympic)	Elite	33	M + F	NA	26.4 (9.8)	6.9 (1.4)	83 (NR)	44 (46)
Mean						25.7 (3.7)	7.3 (0.7)	86 (4)	35 (13)

*ARF* Australian Rules Football, *F* female, *GB* Great Britain, *M* male, *NA* not applicable, *NR* not reported, *SD* standard deviation, *SE* sleep efficiency, *SOL* sleep onset latency, *SS* synchronised swimming, *TST* total sleep time, *WCB* wheelchair basketball

^a^Average number of nights reported

^b^‘Medium threshold’ selected to compute sleep outcomes

^c^Components of the Pittsburgh Sleep Quality Index (PSQI) used to report TST, SOL and SE

### Summary of Sleep Structure and Patterns

Instrumental measurements indicate that while the typical sleep duration of elite athletes may be similar to that of non-athletes, structural differences suggest a more fragmented, lower quality sleep among athletes, with most actigraphy studies reporting athlete sleep efficiencies below 90% (see Table 2). Subjective estimates of sleep duration among athletes are broadly consistent with instrumental measures. Both the duration and structure of sleep showed between-sport and sex differences; total sleep time is shorter in individual (versus team) sports, and also shorter in women.

### Sleep Quality and Insomnia Symptomatology

A total of 12 studies published between 2007 and 2016 reported data on athlete subjective sleep quality and general insomnia symptoms during normal training. While these studies utilised a range of subjective metrics, six used the Pittsburgh Sleep Quality Index (PSQI), a 19-item scale which assesses seven ‘components’ of sleep (sleep quality, sleep efficiency, sleep onset latency, sleep duration, sleep disturbance, daytime dysfunction and sleep medication use), summing the ‘component scores’ to deliver an overall ‘global’ score; global scores >5 indicate ‘poor sleepers’ [66]. Studies reporting general characteristics of sleep quality or PSQI values are presented in Tables 3 and 4, respectively. Data in Table 3 are summarised as prevalence rates of sleep symptoms. Studies administering the PSQI adopted different reporting conventions; both mean scores and threshold (e.g. >5) prevalence rates are therefore shown in Table 4.

**Table 3:** Characteristics of subjective sleep quality in elite athletes

Study	Symptoms assessed	Sport	Level of performance	*n*	Sex	Mean age, y (SD)	Prevalence of symptoms,%		
							Total	M	F
Venter et al. [73]	Experience of sleep problems	Team sports	National	890	M + F	22.3 (3.4)	41	NR	NR
Schaal et al. [69]	Ongoing sleep problems^a^	Multi-sports	National	2067	M + F	23.5 (NR)	22	20	24
Lucidi et al. [68]	Occasional sleep disturbances^b^	Multi-sports	Olympic	103	M + F	23.9 (4.1)	60	59	62
Rodrigues et al. [74]	Sleep dissatisfaction^c^	Para-athletics	Paralympic	40	M + F	30.1 (7.1)	46	NR	NR
Juliff et al. [38]	General sleep disturbance^d^ after a rest period	Multi-sports	Elite	283	M + F	24.1 (5.1)	28	NR	NR
Durán et al. [65]	Insomnia symptoms^e^	Multi-sports (Paralympic)	Elite	33	M + F	26.4 (9.8)	70	NR	NR
Samuels et al. [67]	Abnormal sleep^f^	Multi-sports	Elite	349	NR	NR	13	NR	NR

*F* female, *M* male, *NR* not reported, *SD* standard deviation

^a^Any of sleep onset, sleep maintenance and daytime sleepiness problems in last 6 months

^b^As categorised by the Sleep Disorders Questionnaire[72]

^c^As measured by the Federal University of Sâo Paulo (UNIFESP) Sleep Questionnaire [75]

^d^Any of sleep onset, sleep maintenance, early morning awakening, unrefreshing sleep, or disturbing dreams

^e^Insomnia Severity Index (ISI)

^f^As measured by the Athlete Sleep Screening Questionnaire (ASSQ) [67]

**Table 4:** Sleep assessments in elite athletes using the Pittsburgh Sleep Quality Index (PSQI)

Study	Sport	Level of performance	*n*	Sex	Mean age, y (SD)	Mean global score (SD)^a^	Prevalence, %		
							≥5	>5	>8
Dekker et al. [70]	Gymnastics	National	12	M + F	22.9 (3.5)	6 (NR)	NR	NR	NR
Samuels [1]	Bobsleigh	Elite	24	M + F	27.0 (NR)	6 (1)	78	57	26
Tsunoda et al. [62]	WCB	Elite	14	M	29.5 (5.2)	6 (3)	NR	43	NR
Swinbourne et al. [63]	Team sports	National	175	M + F	21.9 (2.6)	6 (3)	65	50	22
Bleyer et al. [64]	Multi-sports	Elite	452	M + F	21.2 (5.8)	5 (3)	NR	38	NR
Durán et al. [65]	Multi-sports (Paralympic)	Elite	33	M + F	26.4 (9.8)	11 (8)	79	NR	NR
Mean					24.8 (5.4)	7 (4)	74	47	24

*F* female, *M* male, *NR* not reported, *SD* standard deviation, *WCB* wheelchair basketball

^a^Scores of >5 are indicative of clinical sleep disturbance

Overall, the sleep assessments shown in Table 3 show a relatively high level of sleep complaints, with reports of sleep disturbance ranging from 13 to 70%. The low prevalence of ‘abnormal sleep’ reported by Samuels et al. [67] involved an arbitrary cut-off applied to an as-yet unvalidated scale and may not, therefore, represent a robust estimate. In the two studies which reported the prevalence of sleep disturbance by sex [68, 69], rates were highest among women. One study [69] explored this further, reporting that female athletes experienced more problems both initiating and maintaining sleep when compared with their male counterparts. Sleep quality differences between sports were also identified in this study [69], with elite French athletes from aesthetic sports reporting a significantly higher prevalence of insomnia symptoms (33%) compared with all other sports (26% for the sample overall).

Formal (PSQI) assessments of sleep quality suggest similarly high levels of insomnia-type symptoms (Table 4), with mean values at [64] or above the threshold of >5 [1, 62, 63, 65, 70]. This assumption is supported by the prevalence rates reported, with levels of significant sleep disturbance ranging from 38% of multi-sport athletes [64] to 57% of bobsleigh competitors [1]. The more conservative PSQI threshold of >8, indicative of highly disturbed sleep, also showed a relatively high prevalence, ranging from 22 to 26% [1, 63]. However, the possibility that the higher thresholds shown in Table 4 may mask more severe symptoms is suggested by Swinbourne et al. [63], who reported that 9% of elite Australian team sport athletes scored >10. Only one of the studies shown in Table 4 included control comparisons. Tsunoda et al. [62] compared the PSQI global scores of 14 international wheelchair basketball athletes (mean = 6; SD = 3) with 103 non-athletes (mean = 5; SD = 2), and found the difference significant (*p* < 0.05). In the same study, PSQI component score data also showed that athletes reported significantly lower subjective sleep quality and sleep efficiency, even though reported total sleep time showed no significant difference between the groups.

While the PSQI is not a diagnostic tool [66], four studies [30, 65, 68, 69] in this section used instruments validated against insomnia diagnostic criteria which allow inferences to be drawn regarding the prevalence of insomnia cases in elite athlete populations. Using the Athens Insomnia Scale (AIS—an instrument validated against the 10th revision of the International Statistical Classification of Diseases and Related Health Problems criteria for insomnia [71]), Dickinson and Hanrahan [30] reported a mean score for elite multisport athletes of 5 (range 0–16). Since scores of ≥6 indicate clinically significant insomnia symptoms, and since the reported score distribution from this study showed no significant skewness or kurtosis [30], then it can be assumed that, while the study does not report the prevalence of ≥6 scores, a high proportion of athletes must nevertheless have experienced serious insomnia symptoms. Consistent with this assumption, Dickinson and Hanrahan [30] also reported relatively high levels (for this age group) of daytime fatigue among athletes, together with consistent reports (from qualitative interviews) of nonrestorative sleep despite apparently adequate sleep durations. Using the Sleep Disorders Questionnaire (SDQ; a brief questionnaire validated against DSM-IV criteria for insomnia [72]), Lucidi et al. [68] reported that 4% of Italian Olympic athletes met diagnostic criteria for insomnia. Details of symptom chronicity, closely related to insomnia diagnosis, were provided by Schaal et al. [69], who reported a 6-month prevalence of insomnia symptoms of 22%, but a lifetime prevalence of insomnia symptoms of 27%, strongly indicating very high levels of sleep pathology within this nationally representative sample of elite French athletes.

#### Summary of Sleep Quality and Insomnia Symptomatology

The general pattern of results indicates high levels of subjective sleep disturbance and insomnia symptomatology within elite sport, with the evidence suggesting that, within athlete populations, levels of sleep disturbance are higher among women, and among aesthetic athletes. Formal measurements of subjective sleep in athletes also show findings which accord with the objective data, with similarities reported in the total sleep time of athletes and non-athletes, but significantly lower levels of sleep quality reported by athletes. Such evidence, together with that presented in the preceding section, also show that levels of daytime fatigue in athletes can be directly related to degraded night-time sleep.

### Risk Factors for Sleep Disturbance

Studies reporting sleep quality, insomnia symptomatology and changes in sleep patterns broadly focussed on three challenges to athlete sleep: (1) competition (see Tables 5, 6); (2) travel (see Table 7); and (3) training (see Table 8).

**Table 5:** Prevalence of insomnia symptomatology and changes in sleep patterns pre-competition

Study	Sport	Competition (home/away)	*n*	Sex	Mean age, y (SD)	Prevalence of insomnia symptoms (%)	PSQI global mean (SD)	Δ Sleep patterns and sleep quality			
								SE	SOL	TST	SQ
Actigraphy											
Romyn et al. [52]	Netball	National championships	8	F	19.6 (1.5)	NR	NR	↑	↔	↔	↔
Richmond et al. [40]	ARF	AFL match (home)	19	M	24.1 (3.3)	NR	NR	↔	NR	↑	↔
Richmond et al. [39]	ARF	AFL match (home)	10	M	23.0 (2.0)	NR	NR	↔	NR	↑	↔
Shearer et al. [56]	Rugby Union	Celtic League match (home)	28	M	24.4 (2.9)	NR	NR	↔	↔	↔	↔
Fowler et al. [78]	Football	A-League match (home)	6	M	23.4 (NR)	NR	NR	↔	↔	↔	↔
Chennaoui et al. [77]^a^	Swimming	National championships	9	M + F	22.0 (3.0)	NR	NR	↔	NR	↔	NR
Questionnaire											
Elbayoumy and Elbayoumy [79]^b^	Swimming	National championships	40	M	19.0 (1.0)	NR	5 (1)	NR	NR	NR	NR
Swinbourne et al. [63]^b^	Team sports	In competition	75	M + F	NR	NR	6 (3)	NR	NR	NR	NR
Silva and Paiva [76]^b,c^	Gymnastics	FIG World Cup	67	F	18.7 (2.9)	78	7 (3)	NR	NR	NR	↓^b^
Rodrigues et al. [74]^d^	Para-athletics	Paralympic Games	40	M + F	30.1 (7.1)	37	NR	NR	NR	NR	NR
Silva et al. [80]^d^	Para-athletics	Paralympic Games	27	M + F	28.0 (6.0)	70	NR	NR	NR	NR	NR
Erlacher et al. [37]^e^	Multi-sports	Important competition	632	M + F	21.9 (6.8)	66	NR	NR	NR	NR	NR
Juliff et al. [38]^b,e^	Multi-sports	Olympic Games	283	M + F	24.0 (5.0)	64	NR	NR	NR	NR	NR

*AFL* Australian Football League, *ARF* Australian Rules Football, *F* female, *FIG* International Federation of Gymnastics, *M* male, *NR* not reported, *PSQI* Pittsburgh Sleep Quality Index, *SD* standard deviation, *SE* sleep efficiency, *SOL* sleep onset latency, *SQ* sleep quality (subjective rating), *TST* total sleep time, ↑ significant increase, ↔ no significant change, ↓ significant decrease (all *p* < 0.05)

^a^Sleep pattern changes in successful athletes during competition reported only

^b^PSQI with poor sleep quality threshold >5 employed [66]

^c^Comparisons made between successful and unsuccessful athletes during competition

^d^PSQI with poor sleep quality ≥5 employed

^e^Competitive Sports, Sleep, and Dreams questionnaire employed [37]

**Table 6:** Prevalence of insomnia symptomatology and changes in sleep patterns post-competition

Study	Sport	Competition (home/ away)	*n*	Sex	Mean age, y (SD)	Prevalence of insomnia symptoms (%)	Δ Sleep patterns and sleep quality				
							BT	SE	SOL	TST	SQ
Actigraphy											
Shearer et al. [56]	Rugby Union	Celtic League match (home)	28	M	24.4 (2.9)	NR	↑	↔	↔	↓	NR
Fowler et al. [78]	Football	A-League (home)	6	M	23.4 (NR)	NR	↑	↔	↔	↓	↓
Fowler et al. [41]	Football	Pre-season tour (away)	16	M	27.0 (NR)	NR	NR	NR	NR	↓	↓
Fullagar et al. [58]	Football	Pre-FIFA World Cup (away)	15	M	25.5 (4.9)	NR	↑	↔	↔	↓	↔
Richmond et al. [39]	ARF	AFL (home)	10	M	23.0 (2.0)	NR	NR	↔	NR	↓	↓↓
Sleep diaries											
Fullagar et al. [59]	Football	Bundesliga/Eredevisie (home and away)	16	M	25.9 (7.5)	NR	↑	NR	↑	↓	↓
Polysomnography											
Netzer et al. [61]	Cycling	German First Division	13	M	23.9 (NR)	NR	NR	↔	↔	NR	NR
Questionnaire											
Juliff et al. [38]^a^	Multi-sports	Olympic Games	283	M + F	24.0 (5.0)	53	NR	NR	NR	NR	NR

*AFL* Australian Football League, *ARF* Australian Rules Football, *BT* bedtime, *F* female, *M* male, *NR* not reported, *SD* standard deviation, *SE* sleep efficiency, *SOL* sleep onset latency, *SQ* sleep quality (subjective rating), *TST* total sleep time, ↑ significant increase, ↔ no significant change, ↓ significant decrease (all *p* < 0.05); ↓↓ significant decrease (*p* < 0.01)

^a^Ad-hoc question employed: “If you have a late training session or game do you find it hard to sleep after?”

**Table 7:** Changes in sleep patterns, sleep quality and jet lag following long- and short-haul travel

Study	Sport	Flight type (Δ time zone W/E)	*n*	Sex	Mean age, y (SD)	Δ Sleep patterns, sleep quality and JL				
						SE	SOL	TST	SQ	JL
Long-haul										
Actigraphy										
Fullagar et al. [58]^a^	Football	International (4 h W)	15	M	25.5 (4.9)	↔	↔	↔	↔	↑
Fowler et al. [41]^a^	Football	International (1 h W)	16	M	27.0 (NR)	NR	NR	↔	↔	↑
Lastella et al. [57]^a,b^	Football	International (8 h E)	16	M	18.8 (0.9)	↔	NR	↓	↔	NR
Sleep diary										
Fowler et al. [42]^a^	Rugby league	International (11 h W)	18	M	24.2 (3.3)	NR	↔	↑	↔	↑↑
Short-haul										
Actigraphy										
Fowler et al. [78]^c^	Football	Domestic (2 h E and W)	6	M	23.4 (NR)	↔	↔	↔	↔	NR
Richmond et al. [39]^c^	ARF	Domestic (2 h E)	10	M	23.0 (2.0)	↔	NR	↔	↔	NR
Richmond et al. [40]^c^	ARF	Domestic (2 h E)	19	M	24.1 (3.3)	↔	NR	↔	↓	NR

*ARF* Australian Rules Football, *E* eastward travel, *F* female, *JL* jet lag, *M* male, *NR* not reported, *SD* standard deviation, *SE* sleep efficiency, *SOL* sleep onset latency, *SQ* sleep quality (subjective rating), *TST* total sleep time, *W* westward travel, ↑ significant increase, ↔ no significant change, ↓ significant decrease (all *p* < 0.05); ↑↑ significant increase (*p* < 0.01)

^a^Sleep patterns assessed days 1–2 after travel in comparison with pre-travel assessments

^b^Assessments made at low (1600 m) altitude following travel

^c^Assessments made at away matches and compared with home match responses

**Table 8:** Prevalence of insomnia symptoms and changes in sleep patterns during training

Study	Sport	Training	*n*	Sex	Mean age, y (SD)	Prevalence of insomnia symptoms (%)	Δ Sleep patterns and sleep quality				
							RT	SE	SOL	TST	SQ
Training vs rest days											
Actigraphy											
Sargent et al. [33]	Swimming	Olympic preparation	7	M + F	22.5 (1.7)	NR	↓↓	↔	↔	↓↓	NR
Sargent et al. [32]	Multi-sport	Normal training	70	M + F	20.3 (2.9)	NR	↓↓	↔	NR	↓↓	NR
Kölling et al. [60]	Rowing	World Cup preparation	18	M + F	17.7 (0.6)	NR	↓↓	↔	↔	↓↓	↓↓
Questionnaire											
Juliff et al. [38]^a^	Multi-sport	Following a rest day	283	M + F	24.0 (5.0)	28	NR	NR	NR	NR	NR
Intensified vs normal training											
Actigraphy											
Schaal et al. [53]	SS	Olympic preparation	14	F	20.4 (0.4)	NR	↑↑	↓	↑	↓	↔
Kölling et al. [60]	Rowing	World Cup preparation	18	M + F	17.7 (0.6)	NR	↔	↔	↔	↓	↓
Questionnaire											
Juliff et al. [38]^a^	Multi-sport	Heavy training period	283	M + F	24.0 (5.0)	28	NR	NR	NR	NR	NR

*F* female, *M* male, *NR* not reported, *RT* rise time,*SD* standard deviation, *SE* sleep efficiency, *SOL* sleep onset latency, *SQ* sleep quality (subjective rating), *SS* synchronised swimming, *TST* total sleep time, ↑ significant increase, ↔ no significant change, ↓ significant decrease (all *p* < 0.05); ↑↑ significant increase, ↓↓ significant decrease (all *p* < 0.01)

^a^Ad-hoc question employed

#### Competition

Of the studies assessing sleep quality pre-competition, most employed the PSQI (*n * = 7), with five reporting an increased prevalence of complaints (Table 5). Silva and Paiva [76] found that 78% of international female gymnasts scored >5 (indicative of ‘poor’ sleep) on the PSQI prior to an international competition. Gymnasts who scored more ‘competition points’, however, reported significantly worse sleep quality (mean PSQI = 8) than those who scored less (mean PSQI = 6). Sex and inter-sport differences were considered in two studies [37, 38]. Using the Competitive Sports, Sleep and Dreams Questionnaire (CSSDQ), a metric designed to assess sleep habits and disturbances prior to competition [37], two studies reported high prevalence rates of pre-competition sleep disturbance (64–66%), but found no differences between male and female athletes [37, 38]. When comparing sports, however, Erlacher et al. [37] reported a significantly greater frequency of sleep disturbances in individuals (69%) when compared with team sport athletes (60%). Such differences were not supported by Juliff et al. [38], who reported similar levels of sleep disturbance between sports.

Of six studies which used wrist actigraphy to assess sleep patterns prior to competition, most reported no significant changes in sleep efficiency and sleep onset latency when compared with normal training. Two studies, however, reported a significant increase in pre-competition total sleep time [39, 40], while one study [52] reported a significant increase in sleep efficiency. Again, however, there was evidence of sleep–performance relationships. Chennaoui et al. [77] reported that elite swimmers who finished above fourth position at the French national championships exhibited more consistent total sleep times across the competition when compared with swimmers who finished fourth or below, with this latter group reporting significantly longer total sleep times the night before the final race.

The five actigraphy and one sleep diary study shown in Table 6 all reported a significant decrease in total sleep time, and a significantly delayed bedtime, following night competitions, with no study reporting significant changes in sleep efficiency or sleep onset latency. Actigraphy studies assessing post-competition sleep quality more generally were equivocal, with three studies showing a significant decrease [39, 41, 78] and one study showing no change [58]. Using sleep diary assessments, Fullagar et al. [59] also reported a decrease in sleep ‘restfulness’ in elite football players following a night competition, compared with day matches and training days. In the only study assessing PSG-measured sleep structure, Netzer et al. [61] reported a significant increase in stage 3 sleep following competition (compared with rest days) and a significantly increased REM sleep onset latency. However, no changes in sleep onset latency or sleep efficiency were reported.

#### Travel

Studies investigating the impact of both long- (*n* = 4) and short-haul (*n* = 3) travel on athlete sleep are shown in Table 7. Overall, studies used a range of designs and methodological approaches. Three of the studies investigating long-haul travel reported a rating of jet lag in addition to sleep outcomes [41, 42, 58]. Time zone changes ranged from 1 to 11 h, with the maximum time zone change when travelling east being 8 h and the maximum when travelling west being 11 h. Across all studies, however, no change in sleep onset latency or sleep efficiency was reported following travel compared with pre-travel assessments. Changes in total sleep time and sleep quality reported were mixed, with one study showing a significant decrease in total sleep time following long-haul eastward travel [57] and another showing an increase following long-haul westward travel [42]. The majority of studies reported no change in sleep quality; however, Richmond et al. [40] reported a significant decrease in sleep quality prior to away matches following a 2-h eastward time-zone change (mean score = 3.4), when compared with home matches (mean score = 3.8) in elite Australian Rules Football players. Ratings of jet lag following westward travel (no assessments of jet lag were reported following eastward travel) showed a positive trend with increasing time-zone change, with a 1, 4 (both *p* < 0.05) and 11 h time zone (*p* < 0.01) change showing a significant increase from pre-travel assessments.

#### Training

Studies investigating the impact of training on sleep also showed methodological differences, either comparing training days or rest days, or comparing intensified training with normal training. Most studies reported instrumental measures to assess changes in sleep patterns with only one study using a questionnaire. In studies comparing training and rest days, all studies reported significantly earlier rise times and decreased total sleep time on training days (*p* < 0.01). Sargent et al. [32] reported a significant total sleep time gradient relative to training start times across different sports, with earlier start times associated with lower total sleep times and greater pre-training levels of fatigue. Only two studies assessed sleep quality, with one of these reporting a reduction in ‘sleep restfulness’ on a training day compared with a rest day. Three studies quantified levels of daytime sleep [32, 33, 60]. Sargent et al. [32] found elite athletes to nap at similar frequencies on training (15% of 14 days) and rest days (16% of 14 days). However, Kölling et al. [60] reported that the proportion of elite rowers who napped on training days (43%, *n* = 24) was greater than that for rest days (16%, *n* = 9).

In the studies reporting comparisons between intensified and normal training, significant decreases in total sleep time were observed in all actigraphy studies [53, 60]. However, changes in rise time, sleep onset latency, sleep efficiency and sleep quality were equivocal. Schaal et al. [53] reported a significant decrease in sleep efficiency and increase in sleep onset latency, but no change in sleep quality during two weeks of intensified training when compared with baseline values in elite synchronised swimmers. Consistent with this, Juliff et al. [38], using questionnaire assessments, reported that 28% of elite athletes experienced sleep disturbances during periods of heavy training.

#### Summary of Risk Factors

Among elite athletes, predictable events in the training/competition cycle are associated with an increased risk of insomnia symptomatology and disturbed sleep patterns: competition, long- and short-haul travel, and training. Typically, sleep quality significantly declines prior to competition for both men and women. Following competitions, the impact on sleep is related to the timing of events, with late-evening competitions delaying bed times and reducing total sleep time. While the circadian de-synchrony (jet lag) associated with long-haul travel significantly affects sleep patterns, it appears that sleep quality, and instrumental indices of sleep quality such as sleep onset latency and sleep efficiency, are more resilient. Nevertheless, few studies of jet lag or travel fatigue in elite athletes have used formal assessments of insomnia symptoms. Finally, training days can require earlier rise times, with consequent reductions in total sleep time, increased daytime fatigue, and an increased likelihood of daytime napping in some sports.

## Discussion

This review aimed to (1) systematise the research evidence describing sleep patterns and quality among elite athletes and (2) consider the specific risk factors for sleep quality within elite sport. The studies reviewed here broadly support a conclusion that elite athletes experience high levels of sleep disturbance, and that such disturbances are characterised by the symptoms of longer sleep latencies, greater sleep fragmentation, non-restorative sleep, and excessive daytime fatigue. Within elite sport environments, the evidence also suggests that periods of competition, travel and training are likely to precipitate experiences of diminished sleep quality.

### Sleep Patterns

This pattern of degraded sleep quality is clearly illustrated by the composite measure of sleep efficiency derived from the actigraphy studies shown in Table 2. The pooled average sleep efficiency for athletes (mean = 86; SD = 5%), is close to, and for many overlaps, the threshold value of 85%, below which insomnia symptoms are indicated [81]. These experiences are not necessarily associated with shorter sleep durations, with some studies indicating similarly recorded total sleep times in athletes and controls [50, 62]. Despite indications of degraded sleep quality among elite athletes, secondary analyses revealed no significant differences in sleep efficiency in a study comparing full-time female dancers and the Olympic athletes from the study by Leeder et al. [50] during normal training/practice [82]. Such comparisons suggest that low levels of sleep quality may extend to other populations with high levels of expertise, and may not necessarily be exclusive to elite athletes. To some degree, however, differences observed between studies in Table 2 are likely to reflect methodological inconsistencies in instrumental measurements of sleep; in particular, lengths of recording periods selected to establish *normal* sleep patterns, with studies ranging from 3 to 14 nights (see Table 2), and pre-selected thresholds for scoring sleep (e.g. low, medium or high).

### Sleep Quality and Insomnia Symptomatology

Nevertheless, using the single PSQI sleep quality metric, approximately one third to one half of all elite athletes can be categorised as ‘poor sleepers’ (Table 4), with higher levels of insomnia symptoms (up to 70%) reported by Paralympic athletes (Table 3). Despite these apparently high levels of sleep complaints, the evidence does not unequivocally support a conclusion that elite sport *per se* either degrades sleep quality or drives high levels of insomnia diagnosis. Only four studies directly assessed the diagnosis of insomnia. The reported prevalence rate for clinical insomnia among Italian Olympic athletes (4%) [68] falls comfortably within the prevalence range of 3–6% for the general population aged 15–34 years in Southern Europe [83]. Moreover, values reported for the AIS among Australian elite athletes (0–16) [30], while greater than those for healthy adults (0–11) were less than those for diagnosed adult insomniacs (1–24) [84]. The one study which compared athletes and non-athletes found no between-group differences in total sleep time, but did report significantly superior sleep efficiency and sleep quality among non-athletes [62]. To an unknown extent, these outcomes may be influenced by methodological differences. However, while the demands of training, competition and travel certainly contribute to sleep disruption in elite sport, it is also likely that high-performance competitors share sleep vulnerabilities with their non-athlete peers. In general, athletes are drawn from younger high-achieving populations. Epidemiological studies show similarly high levels of sleep disturbance among young people in general, and university students in particular. In a community-based random sample of young adults (aged 18–29 years), for example, Wong and Fielding [85] report 34% scoring >5 on the PSQI, while both Lund et al. [86] and Ye et al. [87] found that 60% of university students were similarly classified by the PSQI as poor sleepers. Given such findings, the expectation, a priori, reflected in the sports science literature that “…poor sleep quality would not be likely in a young, healthy athletic population” [67] is not supported by the epidemiological evidence. Even so, cross-study comparisons do not allow for a definitive judgement to be made on whether elite athletes experience *disproportionately* higher overall levels of insomnia symptoms for their age. Such a judgement requires additional controlled (athlete vs non-athlete) comparisons in the sports science and medicine literature.

### Risk Factors for Sleep Disturbance

The present results broadly identified periods of elevated insomnia symptom risk within training, travel and competition. However, when considered in relation to other achievement-focussed cohorts of younger adults, it does not necessarily follow that “professional sportspeople (both players and officials) face unique challenges relative to their ability to achieve sufficient sleep” [88]. For example, the high prevalence of poor sleep reported prior to competition in elite athletes is similar to that reported by professional ballet dancers prior to a premiere [89] and university students during an exam period [90]. Similarly, the relationship between rise times and total sleep time seen here for elite athletes (e.g. Sargent et al. [32]) is also seen among college students when comparing the impact of earlier (07:00) and later (10:00) class start times [91]. Furthermore, longer total sleep times reported for college students at weekends, when classes were not scheduled [91] are also consistent with studies reporting longer total sleep times on rest days among elite athletes (see Table 8). The rigorous, physical training regimens adhered to by elite athletes is a feature of elite sport which is absent among non-athlete populations. It is also relevant to note, however, that other populations which undertake high levels of physical training or practice, such as performing artists [82, 89], or active-duty military personnel [92], also exhibit high levels of disrupted sleep. The significant reductions in total sleep time and sleep efficiency that were reported during a short-term intensified training period among elite athletes in preparation for an Olympic games [53] were similar to those reported during an extended rehearsal period over 3 months in professional male and female ballet dancers in preparation for a premiere [89]. Given these similarities it is reasonable to suggest that the prevalence of insomnia symptoms in both elite athletes and other groups of younger adults may be mediated by common mechanisms.

States of hyperarousal (manifesting as pre-sleep cognitions and ruminations, stress or worry), were reported to be highly influential in mediating student sleep difficulties [86], and have also been identified as a prime cause of sleep disturbance in elite athletes prior to competition [37, 38]. Lund et al. [86], for example, report that delayed sleep onset (>30 min) was one of the most commonly reported sleep problems among students, with psychometrically assessed ‘stress’ the strongest single predictor of insomnia symptomatology. Similarly, in athlete populations both prior to competition [37, 38] and in general [69], sleep onset problems associated with stress and pre-sleep mentation predominate. Juliff et al. [38] found that the main reasons for delayed sleep onset prior to competitions were “thoughts about the competition” and “nervousness”, while Schaal et al. [69] found that aesthetic athletes, who may be especially concerned with body image and ‘perfection’ in performance, reported the highest levels of stress and sleep onset problems. It is reasonable to conclude, therefore, that cognitive and physiological arousal presents both an explanation for sleep disturbance, and a target for sleep management, among elite athletes.

### Between-Sport Differences

Notwithstanding similarities between *overall* patterns of insomnia symptoms among elite athletes and younger non-athlete populations, it is also clear that some sports impact sleep patterns and quality more than others. Again, levels of pre-sleep arousal appear to play a strong role here, with typical average sleep latencies reported to vary from 40 min (for swimmers) to 8 min (for rugby players) [51]. For example, Schaal et al. [69] described higher levels of sleep complaints reported in aesthetic sports as a result of the ‘psychological toll’ experienced in athletes where success is based upon judgement by others, such as judges and coaches. Conversely, Erlacher et al. [37] suggested team sport athletes may experience less pre-competition anxiety when compared with individual athletes, largely due to a diffusion of responsibility among teams for their competitive outcomes. It is also relevant to note that sex could also explain some of the between-sport differences reported for sleep quality. It is a robust finding in sleep epidemiology that, for all adult age groupings, women tend to report higher levels of insomnia symptomatology than men [83, 85, 86]. Those sports showing higher proportions of female athletes (e.g. aesthetic sports) might be expected to reflect this trend. The evidence reviewed here further suggests that training schedules can contribute substantially to inter-sport differences. Where sports adopt very early training start times (e.g. swimming [33], triathlon, rowing [60]), athletes adopt correspondingly earlier bedtimes [32, 33, 60]. It appears, however, that such adjustment does not always compensate for truncated sleep. As a result, total sleep time tends to be lower and levels of pre-training fatigue and frequency of daytime napping tend to be higher in sports demanding earlier rise times [32, 51]. Training schedules which reduce total sleep time can also have an important, and seemingly paradoxical, impact on sleep structure. Therapeutically, sleep restriction (the induction of a mild state of sleep deprivation) is used in the treatment of insomnia to increase sleep need and thereby reduce sleep onset latencies and increase sleep efficiency (e.g. see Miller et al. [93]). In the actigraphy study by Leeder et al. [50], rowers, who have very early training start times, were also reported to have the lowest total sleep times when compared with canoers, divers and skaters. It is unsurprising, therefore, that rowers were also found to have the shortest sleep onset latencies, and the highest levels of sleep efficiency. Although interpreted as superior sleep quality [50], these latter findings are more likely attributable to restricted sleep.

### Within-Sport Differences

Overall sleep quality and the impact of specific sleep challenges do not appear uniform across athletes. Individual responses to pre-competitive stress, circadian challenges, and late night and early morning scheduling all demonstrate similarly high levels of variance, with some athletes experiencing severe sleep disturbances, while others appear unchallenged. Fullagar et al. [59], for example, reported that, at a squad level, male elite football players experienced diminished total sleep times following late-evening league matches; however, within-squad comparisons also revealed wide individual variations in the degree of resultant sleep deprivation experienced. In this instance, it is possible that, to an unknown extent, these variations could reflect individual differences in chronotype (i.e. positions on the morningness–eveningness continuum) [94], or sleep need (i.e. position on the short sleeper–long sleeper continuum) [94]. In athletes, recent research has indicated a skew towards morning types [1, 80]. However, given the large variations in training schedules adopted across sports, it remains possible that some individual athletes may be disadvantaged by, say, very early training times. Despite the wide range of metrics and instruments employed to identify individuals with sleep pathologies in elite sport (see Table 3), the development of methodologies to explain individual differences in response to sleep challenges, or identification of ‘at risk’ sleep phenotypes remains under researched. For example, in a single study, Juliff et al. [38] suggested that measures of general sleep quality (as measured by the PSQI) were not associated with sleep disturbances experienced during competition periods. This indicates that metrics employed to assess general levels of sleep quality may not necessarily highlight ‘at risk’ phenotypes or inform targeted sleep management in scenarios when individuals are placed under stress (e.g. competition, travel and training).

## Conclusion

While acknowledging the limited number of high quality studies reviewed here, the current literature consistently reports that elite athletes generally show a high overall prevalence of insomnia symptoms characterised by longer sleep latencies, greater sleep fragmentation, non-restorative sleep, and excessive daytime fatigue. These symptoms show marked between-sport differences, with individual sports showing the highest levels of sleep disturbance. Periods of competition and training appear to perpetuate sleep disruptions; however, sleep disruptions reported in response to such sleep challenges exhibit high variability. Two mechanisms in particular are associated with sport-related insomnia symptoms, and therefore offer potential targets for intervention: pre-sleep cognitive/psycho-physiological arousal, and sleep restriction. Evidence is increasing that sleep interventions can improve the quality and extend the duration of athlete sleep [95]. While such outcomes have been reported to enhance aspects of wellbeing, and to a lesser extent performance, further controlled trials are required in this area. Daytime napping appears to be a common compensatory strategy used by athletes; however, there is little evidence that naps are strategically integrated into training regimens. Elite sport could benefit from formal identification of *‘*at risk’ phenotypes to sleep disruption, and subsequent programmes of sleep education and athlete sleep management.

## Electronic supplementary material

Below is the link to the electronic supplementary material.

Supplementary material 1 (DOCX 39.2 kb)

Supplementary material 2 (DOCX 33.9 kb)
