# Supplementary material for: Does Elite Sport Degrade Sleep Quality? A Systematic Review
Source: Sports Med. 2016 Nov 29;47(7):1317–33. doi: 10.1007/s40279-016-0650-6 (PMC5488138; doi:10.1007/s40279-016-0650-6)
Supplement: Supplementary file 1 — Supplementary material 1 (DOCX 39.2 kb) [file 40279_2016_650_MOESM1_ESM.docx]

**Sports Medicine**

**Does elite sport degrade sleep quality? A systematic review**

Luke Gupta,^1,2^ Kevin Morgan,^2^ Sarah Gilchrist^1^

^1^English Institute of Sport, Physiology Department, Bisham, Nr. Marlow, SL7 1RR, UK

^2^Clinical Sleep Research Unit, School of Sport, Exercise and Health Sciences, Loughborough University, LE11 3TU, UK

**Correspondence to**

Luke Gupta

English Institute of Sport, Physiology Department, Bisham, Nr. Marlow, SL7 1RR, UK.

[luke.gupta@eis2win.co.uk](mailto:luke.gupta@eis2win.co.uk)

| **Electronic Supplementary Material Table S1**: Evidence quality appraisal using the Newcastle-Ottawa Scale (NOS) adapted for cross-sectional studies | | | | | | | | | | | | | | | |
| --- | --- | --- | --- | --- | --- | --- | --- | --- | --- | --- | --- | --- | --- | --- | --- |
|  | **Selection**^a^ | | | | | **Comparability**^a^ | | | **Outcome**^b^ | | | **Statistics** | | | |
| Study | Representiveness of the sample | Sample size | Non-respondents | Description of elite athletes | Subtotal | Control for most important factor (athletism) | Control for other factors (Sex and age) | Subtotal | Assessment of outcome | Statistical test | Subtotal | Total (out of 10) | High (>7) | Mod-high (5-7) | Low (<5) |
| Leeder et al.[50] | 0 | 1 | 0 | 1 | 2 | 1 | 1 | 2 | 1 | 1 | 2 | 6 | 0 | 1 | 0 |
| Lastella et al.[51] | 1 | 1 | 0 | 1 | 3 | 0 | 0 | 0 | 2 | 1 | 3 | 6 | 0 | 1 | 0 |
| Richmond et al.[40] | 0 | 1 | 0 | 1 | 2 | 0 | 0 | 0 | 0 | 1 | 1 | 3 | 0 | 0 | 1 |
| Richmond et al.[39] | 0 | 0 | 0 | 1 | 1 | 0 | 0 | 0 | 0 | 1 | 1 | 2 | 0 | 0 | 1 |
| Romyn et al. [52] | 0 | 0 | 0 | 1 | 1 | 0 | 0 | 0 | 2 | 1 | 3 | 4 | 0 | 0 | 1 |
| Schaal et al.[53] | 0 | 1 | 0 | 2 | 3 | 0 | 0 | 0 | 2 | 1 | 3 | 6 | 0 | 1 | 0 |
| Sargent et al.[32] | 1 | 1 | 0 | 1 | 3 | 0 | 0 | 0 | 2 | 1 | 3 | 6 | 0 | 1 | 0 |
| Kölling et al. [60] | 0 | 1 | 1 | 1 | 3 | 0 | 0 | 0 | 2 | 1 | 3 | 6 | 0 | 1 | 0 |
| Fowler et al. [41] | 0 | 0 | 0 | 2 | 2 | 0 | 0 | 0 | 1 | 1 | 2 | 4 | 0 | 0 | 1 |
| Fullagar et al. [59] | 0 | 1 | 0 | 1 | 2 | 0 | 0 | 0 | 1 | 1 | 2 | 4 | 0 | 0 | 1 |
| Fullagar et al. [58] | 0 | 1 | 1 | 1 | 3 | 0 | 0 | 0 | 0 | 1 | 1 | 4 | 0 | 0 | 1 |
| Shearer et al [56] | 0 | 1 | 0 | 1 | 2 | 0 | 0 | 0 | 0 | 1 | 1 | 3 | 0 | 0 | 1 |
| Robey et al. [54] | 0 | 0 | 0 | 1 | 1 | 0 | 0 | 0 | 1 | 1 | 2 | 3 | 0 | 0 | 1 |
| Sargent al. [55] | 0 | 0 | 1 | 1 | 2 | 0 | 0 | 0 | 2 | 1 | 3 | 5 | 0 | 0 | 1 |
| Netzer et al.[61] | 0 | 0 | 0 | 1 | 1 | 0 | 0 | 0 | 1 | 1 | 2 | 3 | 0 | 0 | 1 |
| Juliff et al.[38] | 1 | 1 | 0 | 2 | 4 | 0 | 1 | 1 | 0 | 1 | 1 | 6 | 0 | 1 | 0 |
| Tsunoda et al.[62] | 0 | 0 | 0 | 2 | 2 | 1 | 0 | 1 | 2 | 1 | 3 | 6 | 0 | 1 | 0 |
| Schaal et al.[69] | 1 | 1 | 1 | 2 | 5 | 0 | 1 | 1 | 2 | 1 | 3 | 9 | 1 | 0 | 0 |
| Lucidi et al.[68] | 1 | 1 | 0 | 1 | 3 | 1 | 1 | 2 | 2 | 1 | 3 | 8 | 1 | 0 | 0 |
| Silva et al. [80] | 0 | 1 | 0 | 1 | 2 | 0 | 0 | 0 | 2 | 0 | 2 | 4 | 0 | 0 | 1 |
| Rodrigues et al.[74] | 0 | 1 | 0 | 0 | 1 | 0 | 0 | 0 | 1 | 0 | 1 | 2 | 0 | 0 | 1 |

| **Electronic Supplementary Material Table S1**: Evidence quality appraisal using the Newcastle-Ottawa Scale (NOS) adapted for cross-sectional studies (continued) | | | | | | | | | | | | | | | | |
| --- | --- | --- | --- | --- | --- | --- | --- | --- | --- | --- | --- | --- | --- | --- | --- | --- |
|  | **Selection**^a^ | | | | | | **Comparability**^a^ | | | **Outcome**^b^ | | | **Statistics** | | | |
| Study | Representiveness of the sample | Sample size | | Non-respondents | Description of elite athletes | Subtotal | Control for most important factor (athletism) | Control for other factors (Sex and age) | Subtotal | Assessment of outcome | Statistical test | Subtotal | Total (out of 10) | High (>7) | Mod-high (5-7) | Low (<5) |
| Samuels et al.[67] | 1 | 1 | | 0 | 0 | 2 | 0 | 1 | 1 | 0 | 0 | 0 | 3 | 0 | 0 | 1 |
| Dickinson and Hanrahan [30] | 0 | 0 | | 0 | 1 | 1 | 0 | 0 | 0 | 2 | 1 | 3 | 4 | 0 | 0 | 1 |
| Samuels et al.[1] | 0 | 0 | | 0 | 1 | 1 | 0 | 0 | 0 | 2 | 0 | 2 | 3 | 0 | 0 | 1 |
| Venter et al. [73] | 1 | 1 | | 1 | 1 | 4 | 0 | 0 | 0 | 0 | 1 | 1 | 5 | 0 | 1 | 0 |
| Swinbourne et al. [63] | 1 | 1 | | 0 | 0 | 2 | 0 | 0 | 0 | 2 | 0 | 2 | 4 | 0 | 0 | 1 |
| Bleyer et al. [64] | 1 | 1 | | 1 | 1 | 4 | 0 | 0 | 0 | 2 | 1 | 3 | 7 | 0 | 1 | 0 |
| Chennaoui et al. [77] | 0 | 0 | | 0 | 1 | 1 | 0 | 0 | 0 | 1 | 1 | 2 | 3 | 0 | 0 | 1 |
| Silva and Paiva [76] | 0 | 1 | | 0 | 1 | 2 | 0 | 0 | 0 | 1 | 1 | 2 | 4 | 0 | 0 | 1 |
| Fowler et al. [78] | 0 | 1 | | 0 | 1 | 2 | 0 | 0 | 0 | 1 | 1 | 2 | 4 | 0 | 0 | 1 |
| Erlacher et al. [37] | 1 | 1 | | 0 | 1 | 3 | 0 | 0 | 0 | 1 | 1 | 2 | 6 | 0 | 1 | 0 |
| Fowler et al. [42] | 0 | 0 | | 0 | 1 | 1 | 0 | 1 | 1 | 2 | 1 | 3 | 5 | 0 | 1 | 0 |
| Dekker et al. [70] | 0 | 0 | | 0 | 1 | 1 | 0 | 0 | 0 | 1 | 1 | 2 | 3 | 0 | 0 | 1 |
| Lastella et al. [57] | 0 | 1 | | 0 | 1 | 2 | 0 | 0 | 0 | 1 | 1 | 2 | 4 | 0 | 0 | 1 |
| Durán et al. [65] | 0 | 1 | | 0 | 1 | 2 | 0 | 1 | 1 | 2 | 1 | 3 | 6 | 0 | 1 | 0 |
| Elbayoumy and Elbayoumy [79] | 0 | 1 | | 0 | 0 | 1 | 0 | 0 | 0 | 2 | 1 | 3 | 4 | 0 | 0 | 1 |
| Sargent et al. [33] | 0 | 0 | | 0 | 1 | 1 | 0 | 0 | 0 | 2 | 1 | 3 | 4 | 0 | 0 | 1 |
| **Mean** | **0** | **1** | | **0** | **1** | **2** | **0** | **0** | **0** | **1** | **1** | **2** | **5** | **2** | **12** | **23** |
| **SD** | **0** | **0** | | **0** | **0** | **1** | **0** | **0** | **1** | **1** | **0** | **1** | **2** | **NA** | **NA** | **NA** |
| ^s^ subscale items rated 0-1; ^b^ subscale items rated 0-2; NA, not applicable; SD, standard deviation | | | | | | | | | | | | | | | | |
|  |  | |  |  |  |  |  |  |  |  |  |  |  |  |  |  |
