# Supplementary material for: Does Elite Sport Degrade Sleep Quality? A Systematic Review
Source: Sports Med. 2016 Nov 29;47(7):1317–33. doi: 10.1007/s40279-016-0650-6 (PMC5488138; doi:10.1007/s40279-016-0650-6)
Supplement: Supplementary file 2 — Supplementary material 2 (DOCX 33.9 kb) [file 40279_2016_650_MOESM2_ESM.docx]

**Sports Medicine**

**Does elite sport degrade sleep quality? A systematic review**

Luke Gupta,^1,2^ Kevin Morgan,^2^ Sarah Gilchrist^1^

^1^English Institute of Sport, Physiology Department, Bisham, Nr. Marlow, SL7 1RR, UK

^2^Clinical Sleep Research Unit, School of Sport, Exercise and Health Sciences, Loughborough University, LE11 3TU, UK

**Correspondence to**

Luke Gupta

English Institute of Sport, Physiology Department, Bisham, Nr. Marlow, SL7 1RR, UK.

[luke.gupta@eis2win.co.uk](mailto:luke.gupta@eis2win.co.uk)

| **Electronic Supplementary Material Table S2**: the modified Swann et al.’s taxonomy of ‘eliteness’ | | | | | | | |
| --- | --- | --- | --- | --- | --- | --- | --- |
|  | With-in sport | | | Between sport | | Statistics | |
|  | A | B | C | D | E |  |  |
| Study | Standard of performance | Success at level | Experience at level | Competiveness in country | Global competitiveness of sport | Scores (out of 16) | Competitive Elite (>8) |
| Leeder et al.[50] | 4 | NR | NR | 2 | 3 | 10 | 1 |
| Lastella et al.[51] | 4 | NR | NR | 2 | 3 | 10 | 1 |
| Richmond et al.[40] | 4 | NR | NR | 4 | 2 | 12 | 1 |
| Richmond et al.[39] | 4 | NR | NR | 4 | 2 | 12 | 1 |
| Romyn et al. [52] | 2 | NR | NR | 2 | 3 | 5 | 0 |
| Schaal et al.[53] | 4 | NR | 3 | 2 | 2 | 8 | 0 |
| Sargent et al.[32] | 4 | NR | NR | 2 | 3 | 10 | 1 |
| Kölling et al. [60] | 3 | NR | NR | 1 | 4 | 8 | 0 |
| Fowler et al. [41] | 4 | NR | NR | 3 | 2 | 10 | 1 |
| Fullagar et al. [59] | 4 | NR | NR | 4 | 4 | 16 | 1 |
| Fullagar et al. [58] | 4 | NR | 3 | 4 | 4 | 16 | 1 |
| Shearer et al [56] | 4 | NR | NR | 4 | 4 | 16 | 1 |
| Robey et al. [54] | 2 | NR | NR | 2 | 4 | 6 | 0 |
| Sargent al. [55] | 2 | NR | NR | 1 | 4 | 5 | 0 |
| Netzer et al.[61] | 2 | NR | NR | 1 | 4 | 5 | 0 |
| Juliff et al.[38] | 4 | NR | 4 | 2 | 4 | 12 | 1 |
| Tsunoda et al.[62] | 4 | NR | 4 | 1 | 3 | 8 | 0 |
| Schaal et al.[69] | 4 | NR | 2 | 2 | 2 | 8 | 0 |
|  | | | | | | | |

| **Electronic Supplementary Material Table S2**: the modified Swann et al.’s taxonomy of ‘eliteness’ (continued) | | | | | | | |
| --- | --- | --- | --- | --- | --- | --- | --- |
|  | With-in sport | | | Between sport | | Statistics | |
|  | A | B | C | D | E |  |  |
| Study | Standard of performance | Success at level | Experience at level | Competiveness in country | Global competitiveness of sport | Scores (out of 16) | Competitive elite (>8) |
| Lucidi et al.[68] | 4 | NR | NR | 2 | 3 | 10 | 1 |
| Silva et al. [81] | 4 | NR | NR | 1 | 3 | 8 | 0 |
| Rodrigues et al.[74] | 4 | NR | NR | 1 | 3 | 8 | 0 |
| Samuels et al.[67] | 4 | NR | NR | 4 | 3 | 14 | 1 |
| Dickinson and Hanrahan [30] | 4 | NR | NR | 2 | 2 | 8 | 0 |
| Samuels et al.[1] | 4 | NR | NR | 4 | 3 | 14 | 1 |
| Venter et al. [73] | 4 | NR | NR | 4 | 4 | 16 | 1 |
| Swinbourne et al. [63] | 4 | NR | NR | 4 | 4 | 16 | 1 |
| Bleyer et al. [64] | 3 | NR | NR | 1 | 3 | 6 | 0 |
| Chennaoui et al. [77] | 4 | 3 | NR | 1 | 4 | 10 | 1 |
| Silva and Paiva [76] | 4 | NR | 4 | 3 | 4 | 14 | 1 |
| Fowler et al. [78] | 4 | NR | NR | 4 | 2 | 12 | 1 |
| Erlacher et al. [37] | 4 | NR | NR | 2 | 2 | 8 | 0 |
| Fowler et al. [42] | 4 | NR | NR | 3 | 2 | 10 | 1 |
| Dekker et al. [70] | 2 | NR | NR | 1 | 3 | 4 | 0 |
| Lastella et al. [57] | 3 | NR | NR | 3 | 4 | 11 | 1 |
| Durán et al. [65] | 4 | NR | NR | 1 | 3 | 8 | 0 |
| Elbayoumy and Elbayoumy [79] | 3 | NR | NR | 1 | 3 | 6 | 0 |
| Sargent et al. [33] | 2 | NR | NR | 1 | 3 | 4 | 0 |
| **Mean** | **3** | **NA** | **NA** | **2** | **3** | **10** | **20** |
| **SD** | **1** | **NA** | **NA** | **1** | **1** | **4** | **NA** |
| Modified equation,[A x ((D + E)/2)]; a score >8 is judged to be a study that has recruited ‘competitive elite’ athletes (see Swann et al. [46]); NR, not reported; NA, not applicable; SD, standard deviation | | | | | | | |
